# Cold stress induces colitis-like phenotypes in mice by altering gut microbiota and metabolites

**DOI:** 10.3389/fmicb.2023.1134246

**Published:** 2023-04-11

**Authors:** Lijuan Sun, Xueying Wang, Yuankang Zou, Yixuan He, Changting Liang, Juan Li, Pu Li, Jianbin Zhang

**Affiliations:** ^1^Key Laboratory of Resource Biology and Biotechnology in Western China, Ministry of Education, School of Medicine, Northwest University, Xi’an, China; ^2^Department of Occupational and Environmental Health and the Ministry of Education Key Lab of Hazard Assessment and Control in Special Operational Environment, School of Public Health, Fourth Military Medical University, Xi’an, China; ^3^Department of Anesthesiology, The Second Affiliated Hospital of Air Force Medical University, Xi’an, Shanxi, China

**Keywords:** cold stress, inflammatory bowel disease, gut microbiota, gut–brain axis, animal research

## Abstract

**Introduction:**

The modernized lifestyle has been paralleled by an epidemic of inflammatory bowel disease (IBD). Excessive consumption of cold beverages is especially common among the modern humans. However, whether cold stress contributes directly to the gut barrier and gut–brain axis is not clear.

**Methods:**

We conducted a cold stress model induced by cold water. The mice were treated with 14 consecutive days of intragastric cold or common water administration. We observed changes in gut transit and gut barrier in the colon. We also employed RNA sequencing-based transcriptomic analysis to identify the genes potentially driving gut injury, and simultaneously examined the gut microbiota and metabolites in the feces.

**Results:**

We found that cold stress disturbed the intestinal function and increased gut permeability. A set of core genes related to immune responses were consistently overexpressed in the cold stress group. Additionally, cold stress induced decreased bacterial diversity, ecological network, and increased pathogens mainly belonging to Proteobacteria. The dopamine signaling pathway-related metabolites were largely reduced in the cold stress group.

**Conclusion:**

This study revealed that cold stress could trigger an IBD-like phenotype in mice, implying that cold stress is a possible risk factor for IBD development.

## Introduction

The modernized lifestyle has been paralleled by an epidemic of several gastrointestinal diseases worldwide beginning in the 1980s, including irritable bowel disease (IBD) characterized by gut barrier dysfunction (Ananthakrishnan, 2015; Khademi et al., 2021). Especially, the incidence of IBD is continuously rising among young people, even among children (Barnes and Kappelman, 2018). Patients with IBD present symptoms similar to those of patients with irritable bowel syndrome (IBS) to some extent, including anxiety and depression (Spiller and Major, 2016). These findings suggest a potential link between modernized lifestyle, gut barrier, and gut–brain interaction.

Excessive consumption of cold beverages is highly prevalent in modern population subsets, especially in the youth. Mounting evidence has shown that sweeteners, food additives, and other food ingredients in beverages play important roles in IBD (He et al., 2021). However, whether some other related factors (for instance, cold stress in the gut) might affect IBD occurrence or not, is still not quite clear. It has shown that cold meal intake disturbs contractile activity of the gastric (Sun et al., 1995; Verhagen et al., 1998).

Interestingly, industrial and domestic refrigeration development is regarded as a key environmental factor in the etiology of IBD since it increases the chance for exposure of human populations to psychrotrophic bacteria (such as *Yersinia*), which exacerbate intestinal inflammation (Hugot et al., 2021). However, only approximately 10% of patients with IBD were found to harbor *Yersinia* in their gut (Le Baut et al., 2018); therefore, the theory of psychrotrophic bacteria cannot explain these conditions completely. Consequently, some other related factors (for instance, cold stress in the gut) might affect IBD occurrence.

To untangle the link between cold stress, gut barrier, and the gut–brain interaction, we mimicked excess cold beverage consumption in a mouse model. Metabolic syndrome is a potentially important confounder factor, which can indirectly affect the gut–brain axis by changing a myriad of physiological and endocrine systems (Sun et al., 2018). To uncouple the metabolic effects directly caused by excess calorie intake from the beverage rather than the cold stress, we treated mice with cold water by gavage for 14 consecutive days. We then evaluated the gastrointestinal physiology and stress-related behavior in mice. Moreover, we investigated whether cold stress could affect the microbiota and metabolites of fecal contents since gut microbiota and metabolites are essential for bidirectional interactions within the gut–brain axis. These findings could highlight a link between cold stress on gut barrier dysfunction, gut microbiota, and metabolite disturbance, providing new preventive strategies to quell the creeping up in the incidence of IBD.

## Materials and methods

### Animals

The C57BL/6 mice (8 weeks, male) were used in this study. Sixteen mice were divided into two groups: In the control group (n = 8), the mice were subjected to gavage with room-temperature water (20–25°C). In the cold stress group (n = 8), the mice were subjected to gavage with cold water (0–4°C).

Regular drinking water and food were available to the mice at all times. Additionally, each mouse was given a total of 1 ml cold or room-temperature water. Prior to use the water was autoclaved and handled using sterile vessels. The maximum dose given by gavage was 200 μl at a time, and we gave it five times. This treatment had lasted for 2 weeks. Gut function tests and behavioral tests were carried out after gavage for 2 weeks. The protocol was approved by the Animal Research Ethics Board of the Northwest University (Approve number: 20210204).

### Fecal output, fecal moisture content evaluation, and Bristol score

All the mice were transferred into separate clean cages, and the feces were numbered and collected for 4 h between 8 am, and 12 am. All the feces during the procedure were collected and placed in tubes, then tubes were weighed and the wet weight were recorded, dried overnight at 60°C, then reweighed, and the dry weight were recorded. At last the water content was calculated. The criteria for Bristol score were evaluated as described in the previous study (Koh et al., 2010).

### Histological analysis and disease activity index score

The mice were killed by cervical dislocation. Then, the mice were dissected and the whole intestines were removed from the abdomen. Several segments were taken from the ileum and colon, respectively, and rinsed with cold saline, fixed in 4% polyformaldehyde. The ileum and colon samples were prepared for histological examination of lesions. The resected segments were opened lengthwise, embedded in paraffin, and sectioned. Hematoxylin–eosin staining of the ileum and colon was performed. Intestinal inflammation was assessed by observing ulcer formation, epithelial cell changes (goblet cells), lymphocyte infiltration, and lymph node formation, according to a previous report (Zielinska et al., 2017). DAI score is evaluated according the stool consistency, blood and weight loss as previously described (Kim et al., 2012).

### Gut peristalsis

In brief, 0.6 g of carmine was dissolved in 0.5% hydroxymethyl cellulose solution to obtain a 6% carmine solution. A 200 μl aliquot of the 6% carmine solution was administered to the mice *via* gavage. The animals were then placed in separate cages on a white sheet of paper (to facilitate recognition of red feces). Gut peristalsis activity was defined as the time between gavage and the first red bolus excreted (Zielinska et al., 2017).

### Indirect calorimetry

In this study, we randomly selected 6 mice from the control group and 6 mice from the cold stress group, to perform the metabolic tests. Twelve mice were transferred into metabolic cages (Columbus Comprehensive Lab Animal Monitoring System, Columbus, OH, USA) and housed for 24 h with food and water provided *ad libitum*. After 24 h of adaptation, the oxygen volume (VO2), carbon dioxide volume (VCO2), respiratory exchange ratio (RER; VCO2/VO2), heat production, food intake, and drink intake were automatically measured during the following 24 h by the system.

### Immunofluorescence

The immunofluorescence was performed on paraffin section from the colon tissue as described (Dinallo et al., 2019). The slides were incubated overnight at 4°C with the primary antibodies E-cadherin (1:1000; BD Biosciences) and ZO-2 (1:1000; Cell Signaling Technology), after washing three times with PBS, slides were incubated for 1 h at room temperature with specific secondary antibodies coupled with Alexa Fluor Dyes (1:300; Servicebio). Coverslips were mounted on glass slides using ProLong Gold antifade reagent with DAPI to counterstain the DNA. Lastly, the images were acquired on fluorescence microscope.

### Depression- and anxiety-like behaviors test

The tail suspension test (TST) was used to evaluate depression- like behaviors. As described previously (Steru et al., 1985), the mice were admitted to suspend for 6 min. All sessions were video-recorded. The time spent struggling in TST (The mice have obvious struggling movements) within a 6-min session was recorded and evaluated as behaviors for survival. The behaviors with reduced time for struggling were regarded as depression-like behaviors. Additionally, anxiety-like behaviors were examined using Open Field Test (OFT) as described previously (Walsh and Cummins, 1976). Their locomotor activity were monitored for 15 min. The time spent in the central area were recorded as indicators of exploratory behavior. The behaviors with reduced time in the center were regarded as anxiety-like behaviors.

### Feces collection, 16S rDNA sequencing, and untargeted metabolomic analysis

Feces samples were collected between 8 am and 11 am. The feces samples collected were stored at −80°C. The feces DNA extraction, polymerase chain reaction, library construction, and sequencing were performed as previously described (Sun et al., 2019a). Diversity indices were calculated using QIIME2. Principal coordinate analysis was performed using Calypso online tools. The relative abundances, Spearman correlation coefficients, and heatmap were calculated and compared using T packages. Spearman’s rank correlations at the genus level were calculated as our previous study (Sun et al., 2019b).

The liquid chromatography-mass spectrometry (LC–MS) was employed to analyze the fecal metabolome. We used the ACQUITY UPLC system (Waters Corporation, Milford, MA, United States) coupled with an AB SCIEX Triple TOF 6600 System (AB SCIEX, Framingham, MA, United States) in positive- and negative-ion modes as described previously (Su et al., 2020; Yan et al., 2020).

### RNA sequencing and bioinformatics analysis

The TRIzol reagent (Invitrogen, Carlsbad, CA, United States) was used to extract the RNA, then assess the RNA integrity by Bioanalyzer 2,100 (Agilent, CA, United States). Poly(A) RNA was purified and fragmented into small pieces, and reverse-transcribed to synthesize cDNA, treated with U-labeled double-stranded DNAs with the heat-labile enzyme UDG, then amplified. Finally, the products were sequenced on an Illumina NovaSeq 6,000 platform (LC-Biotechnology Co., Ltd., Hangzhou, China).

We used the Fastp software for quality control, used HISAT2 to map the reads, and used StringTie to assemble them. The different mRNAs were selected as the following criteria: mRNAs with fold change of >2 or < 0.5 and *p* < 0.05 in a parametric F-test comparing nested linear models.

### Statistical analysis

All data in gut function and behavioral tests were showed as the mean ± SEM. Statistical analysis was performed by using Student’s t-test for comparisons between cold stress group and control group. Statistical analysis for bioinformatics data of RNA-seq, gut microbiota, and metabolites was performed using the R package vegan. The *p* value less than 0.05 was regarded as statistical significance.

## Results

### Cold stress disturbs intestinal function and increases gut permeability in mice

**Figure 1:**
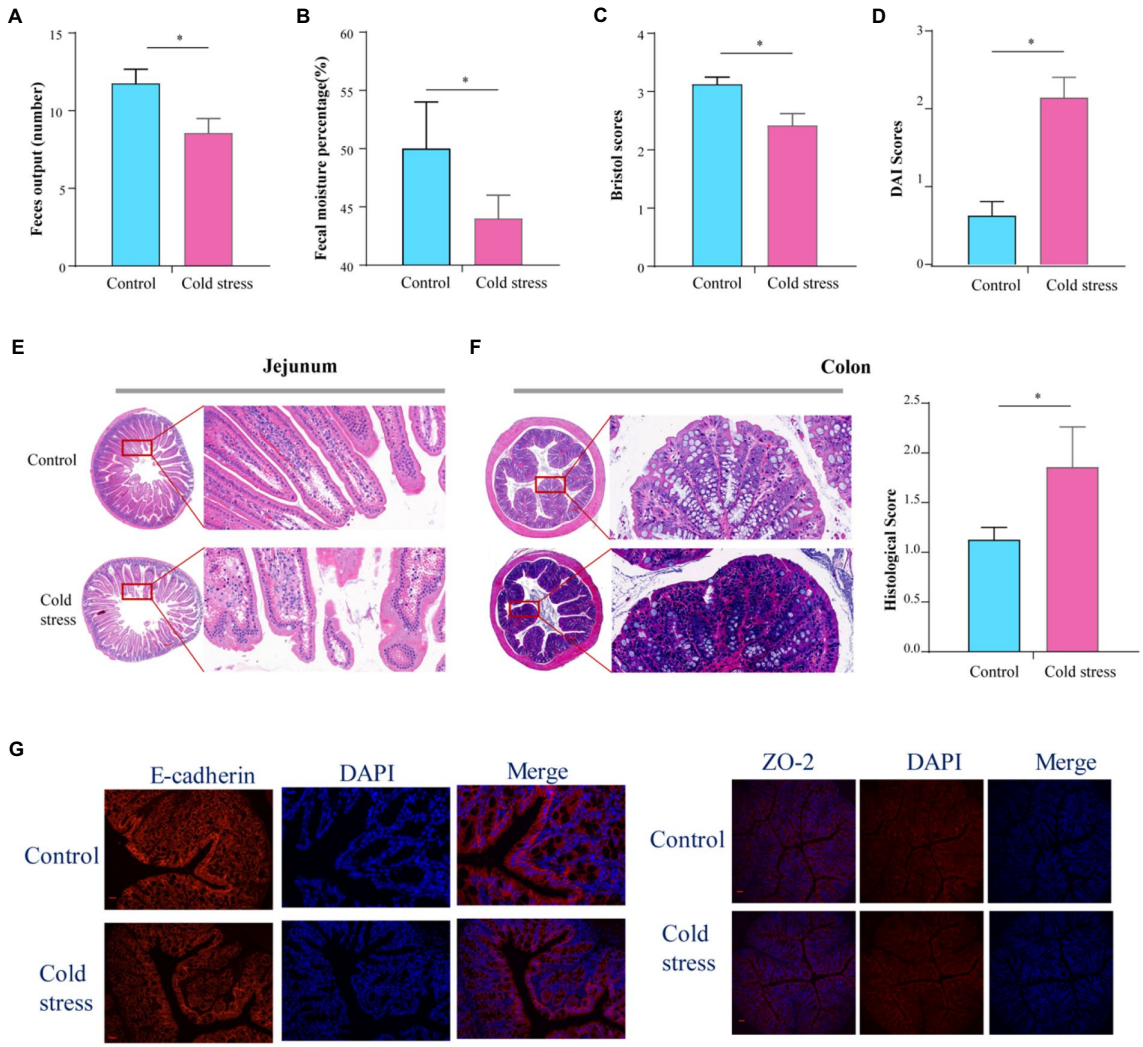
Cold stress disturbs intestinal function and increases gut permeability in mice. **(A)** Feces output in 4 h. **(B)** Fecal moisture percentage. **(C)** Bristol scores. **(D)** DAI scores calculating using body weight and fecal characters. **(E,F)** Hematoxylin and eosin-stained jejunum and colon sections from control and cold stress-treated mice and the corresponding histological scores. **(G)** Representative images of markers of gut barrier in the colon, E-cadherin (left) and ZO-2(right) Scale bars = 50 μm. All the data are expressed as mean ± SEM (*n* = 8 in the control group; *n* = 7 in the cold stress group). *p < 0.05.

A total of 15 mice (8 in control group and 7 in cold stress group) were able to complete the experiment, as one mouse died from choking during gavage. There were no significant differences in heat production, food intake, drink intake, or respiratory exchange rate between the two groups (Supplementary Figures S1A–D). No changes were observed in the jejunum (Supplementary Figure S1E) or colon length (Supplementary Figure S1F) in the cold stress group. Assessment of gastrointestinal motility by carmine red administration showed that gastrointestinal propulsion was impaired in the cold stress group (Supplementary Figure S1G). In the cold stress group, lower fecal output (*t* = 2.493, *p* = 0.027; Figure 1A), lower fecal moisture percentage (*t* = 4.815, *p* < 0.001; Figure 1B), and lower Bristol scores (*t* = 3.015, *p* = 0.010; Figure 1C) were observed. These results suggest that cold stress disturbs the intestinal function. Altered fecal properties, bloody stools, and weight loss are the most important characteristics in IBD, then we used the DAI scores to evaluate the severity of the colitis. We found that the DAI scores in the cold stress group were significantly higher than in the control group (*t* = −4.861, *p* < 0.001; Figure 1D). Correspondingly, hematoxylin–eosin staining showed sparse intestinal villus, and edema in the intestinal villi of the jejunum (Figure 1E), a significant reduction in goblet cells, an enhanced inflammation response, and an elevated histological score in the colon (*t* = −2.193, *p* = 0.047; Figure 1F). As to the gut barrier, we found that the expression of E-cadherin and ZO-2 was lowered by immunofluorescence (Figure 1G), compared to that in the control group.

### Cold stress exacerbates the inflammatory response of the intestinal tissue in mice

**Figure 2:**
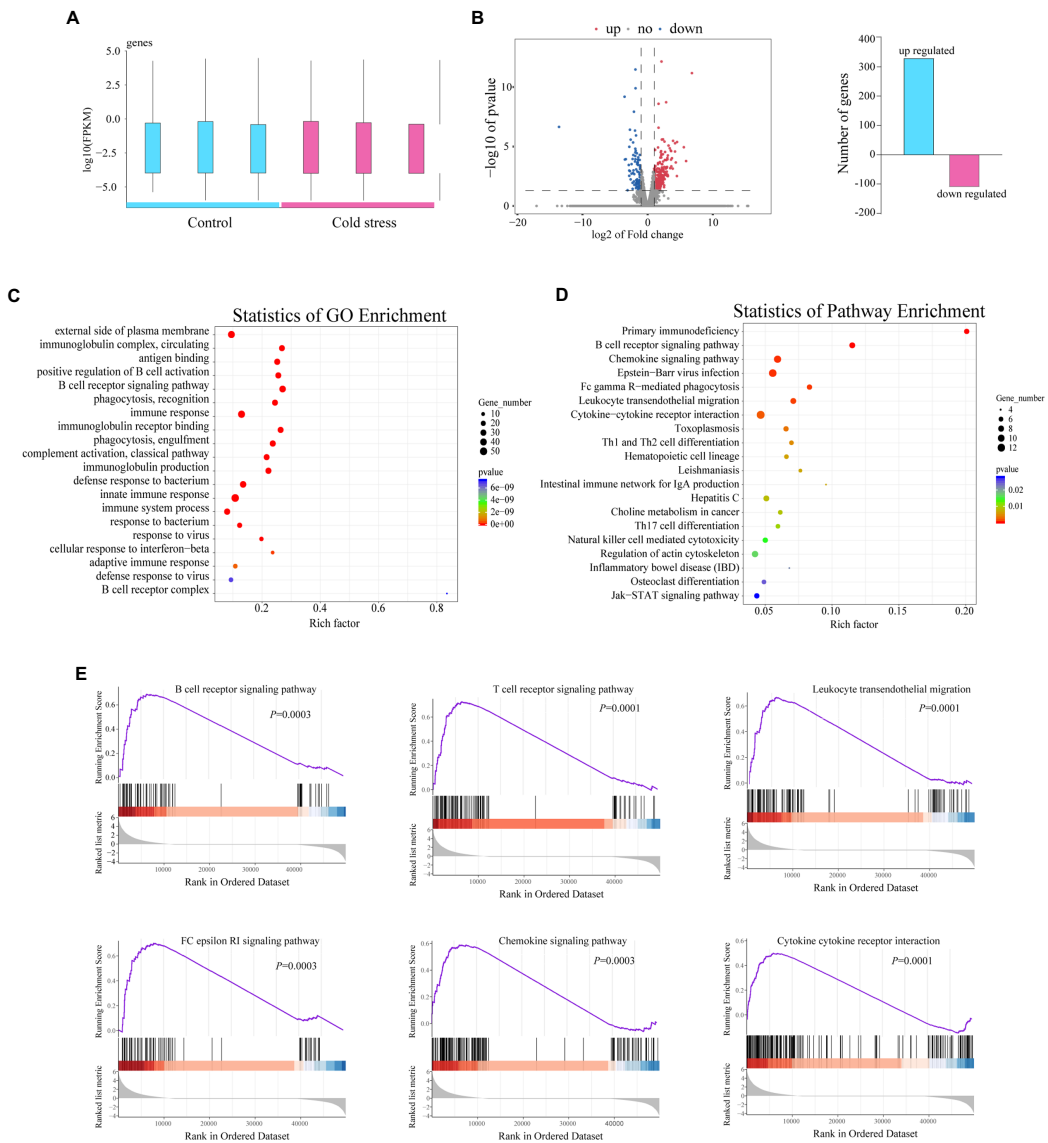
Cold stress exacerbates the inflammatory response of the intestinal tissue in mice. **(A)** Number of genes upregulated and downregulated in the cold water-treated group. **(B)** Volcano plot of genes differentially expressed between the two groups. **(C,D)** GO and KEGG pathway enrichment analysis between the two groups. **(E)** Gene set enrichment analysis of the cold stress group compared to the control group. Data are representative of three biological repeats (*n* = 3).

To further identify the changes in gene expression in the intestinal tissue after cold stress, we randomly selected out 6 mice from the two groups and investigated the gene expression profiles of the intestinal tissue using RNA sequencing (Figure 2A). A total of 432 genes with differential expression between groups were identified, with 330 genes upregulated and 102 genes downregulated in the cold stress group when compared with the control group (Figure 2B). Gene Ontology analysis showed that all the genes most strongly enriched in immune function processes, including innate immune response and adaptive immune response, such as the B cell activation and B cell receptor complex, immunoglobulin production, immunoglobulin complex, complement activation, antigen binding, and defense response to a bacterium (Figure 2C). In accordance with these findings, KEGG pathway analysis also showed that the genes were enriched in immune activation in the colon, including the primary immunodeficiency, B cell receptor signaling, intestinal immune network for IgA production, NK cell-mediated cytotoxicity pathways, leukocyte transendothelial migration, Th1 and Th2 cell differentiation, Th17 cell differentiation, cytokine–cytokine receptor interaction, and chemokine and Fc gamma R-mediated phagocytosis, all of which are closely related to the immune response in the colon (Figure 2D).

To further reveal the effects of cold stress on the colon, we used gene set enrichment analysis to analyze the genes which revealed substantial upregulation of genes involved in the B and T cell receptor signaling pathway, leukocyte transendothelial migration, FC epsilon RI signaling pathway, chemokine signaling pathway, and cytokine–cytokine receptor interaction (Figure 2E). These findings suggested that cold stress triggers an IBD-like phenotype in mice.

### Cold stress leads to low bacterial diversity and a fragile ecological network in the gut microbiota

In general, IBD is considered to occur when the immune system overreacts to the resident gut microbiota, inducing a chain of inflammatory events that can destroy the gut barrier (Eisenstein, 2016). These findings prompted us to further investigate whether changes in microbiota or bacterial metabolites from the feces under cold stress stimulation may regulate the gut barrier and gut–brain interactions.

**Figure 3:**
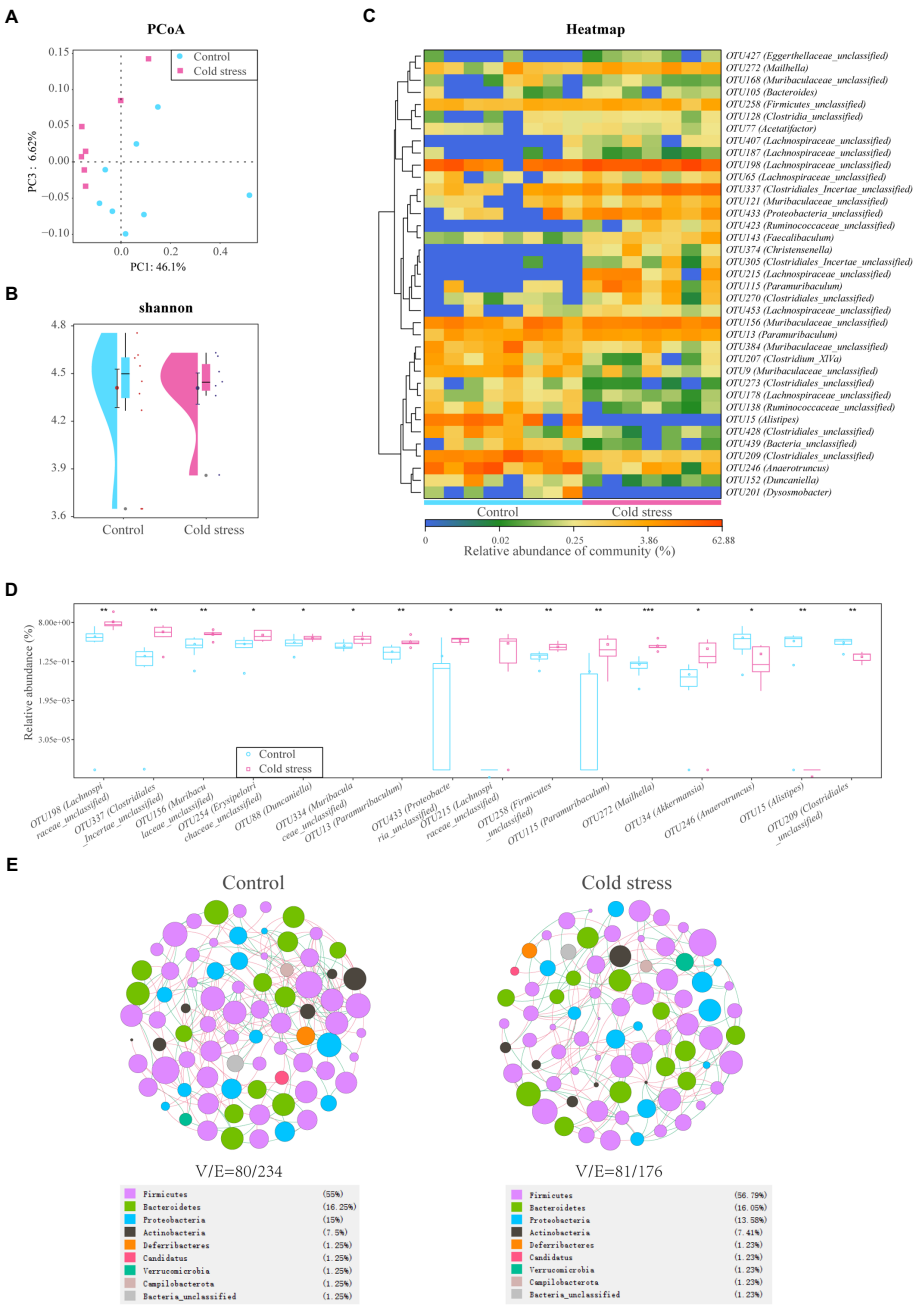
Cold stress leads to low bacterial diversity and a fragile ecological network in the gut microbiota. **(A)** Principal component analysis (PCoA) plots of unweighted UniFrac distances between the two groups. **(B)** Shannon diversity scores. **(C)** Heatmap of key operational taxonomic units (OTUs). **(D)** Gut bacteria with different abundances at the OTU level. **(E)** Network analysis at the genus level. V: number of nodes. E: number of edges. (*n* = 8 in control group and *n* = 7 in cold stress group).

Eight individual fecal samples from the control group and seven individual fecal samples from the cold stress group were collected and sequenced. The principal component plots with unweighted UniFrac distances showed a clear separation between the cold stress and control groups (Figure 3A), which suggested that cold stress led to a significant alteration in the gut microbiota composition. The Shannon index showed no difference between the two groups (Figure 3B). However, at the operational taxonomic unit (OTU) level, the microbiota were significantly differed (Figure 3C). The abundances of *OTU198* (*Lachnospiraceae_unclassified*), *OTU337* (*Clostridiales_Incertae_unclassified*), *OTU156* (*Muribaculaceae_unclassified*), *OTU254* (*Erysipelotrichaceae_unclassified*), *OTU88* (*Duncaniella*), *OTU334* (*Muribaculaceae_unclassified*), *OTU13* (*Paramuribaculum*), *OTU433* (*Proteobacteria_unclassified*), *OTU215* (*Lachnospiraceae_unclassified*), *OTU258* (*Firmicutes_unclassified*), *OTU115* (*Paramuribaculum*), *OTU272* (*Mailhella*), and *OTU34* (*Akkermansia*) were significantly increased in the cold stress group. Additionally, the abundances of *OTU246* (*Anaerotruncus*), *OTU15* (*Alistipes*), and *OTU209* (*Clostridiales_unclassified*) decreased under cold stress (Figure 3D). The detailed information has been shown in Supplementary Table S1. Linear discriminant analysis of effect size further showed that the bacteria with increased abundance in the cold stress group mainly belong to Proteobacteria (Supplementary Figure S2). To determine the pattern of bacteria, we constructed their networks in the two group respectively, we found that the network of the cold stress-treated mice had a simpler property (nodes/edges = 81/176) than the control group (nodes/edges = 80/234), indicating that cold stress may induce vulnerability to environmental stress in the gut microbiota (Figure 3E).

### Cold stress downregulates metabolites of the dopamine-related pathway in the intestinal flora

**Figure 4:**
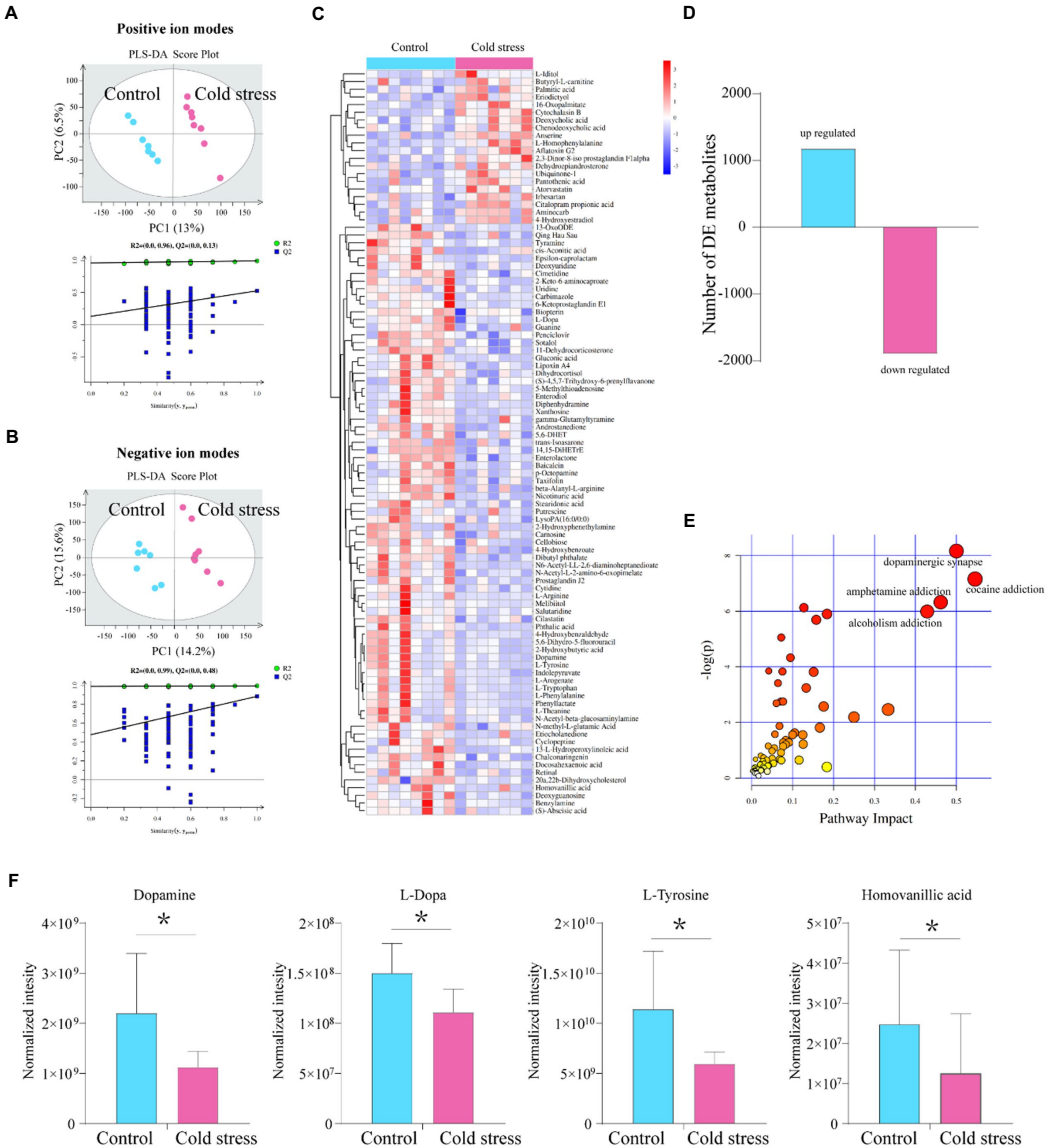
Metabolites in the dopamine-related pathway are downregulated by cold stress treatment. **(A)** Partial least-squares discriminant analysis (PLS-DA) scores of all peak features in positive-ion mode. **(B)** PLS-DA scores of all peak features in negative-ion mode. **(C)** Heatmap of the metabolites that altered in the cold stress group. **(D)** Number of differentially expressed (DE) metabolites between the two groups. **(E)** Pathways impacted. **(F)** Metabolites in dopamine-related pathways (dopamine, l-dopamine, l-tyrosine, and homovanillic acid). *p < 0.05 (*n* = 8 in control group and *n* = 7 in cold stress group).

As a microbe–host bridge, some metabolites of the intestinal flora can affect host physiology by entering the bloodstream. Therefore, we analyzed fecal metabolites using LC–MS. We found that the metabolic data clusters of the control and cold stress groups were separated from each other in both positive- and negative-ion modes by partial least-squares discriminant analysis (Figures 4A,B). The heatmap also showed that cold stress led to significant alterations in fecal metabolite levels (Figure 4C); 1,179 metabolites were upregulated, and 1896 metabolites were downregulated with significant changes (Figure 4D). The most strongly impacted metabolic pathways included cocaine addiction, dopaminergic synapse, amphetamine addiction, and alcoholism addiction (Figure 4E), all of which were accompanied by a significant reduction in levels of dopamine, l-dopamine, l-tyrosine, and homovanillic acid (Figure 4F). As one of the most important neurotransmitters, decreased dopamine levels may contribute to anxiety-like and depression-like behaviors in the cold stress mice. Additionally, patients with IBD present some similar symptoms to patients with IBS, including anxiety and depression. Therefore, these behaviors were further evaluated.

### Cold stress increases depression-like behaviors in mice

**Figure 5:**
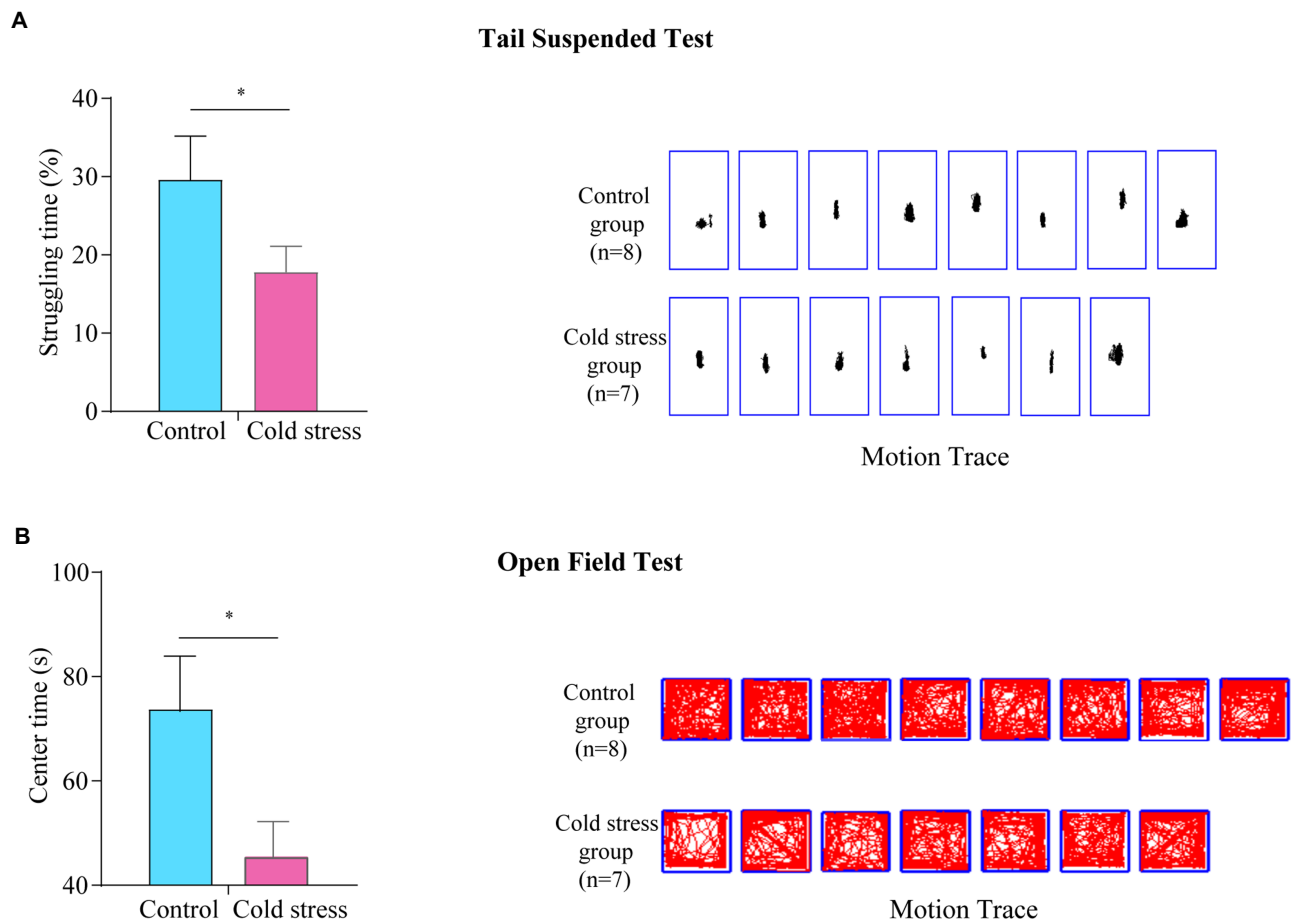
Cold stress increases anxiety- and depression-like behaviors. **(A)** Struggling time (left) and motion tracking (right) in the tail suspended test. (B) Center time and motion tracking in the open field test. *p < 0.05. (*n* = 8 in control group and *n* = 7 in cold stress group).

In the tail suspended test, the struggling time was significantly decreased in the cold stress group (*t* = 2.347, *p* = 0.035; Figure 5A), suggesting an increase in depression-like behaviors in mice exposed to cold stress. Furthermore, in the open field test, the center time (*t* = 2.451, *p* = 0.029) was reduced in the cold stress group (Figure 5B), implying a tendency of decreased exploratory behavior and increased anxiety-like behavior in the cold stress group.

### Correlations of gut microbiota and metabolic changes

Finally, to explore the functional significance of the metabolite perturbations in the gut microbiota of the cold stress-treated group, the 97 annotated metabolites with significant differences were selected, and their Spearman correlation coefficients with different bacteria were calculated. Significant correlations were observed between the gut microbiota and metabolites (Supplementary Figure S3A), and also observed between metabolites and gut function (Supplementary Figure S3B).

## Discussion

Increased beverage consumption has long been recognized to play an important role in the pathogenesis of metabolic diseases and colorectal cancer (Yoshida and Simoes, 2018; Goncalves et al., 2019; Hur et al., 2021). Although food additives such as emulsifiers, food colorants, titanium dioxide, and aluminum have largely contributed to the development of these conditions, recent evidence have also identified that food additives are pathogenic factors for colitis (He et al., 2021). In this study, we focused on the potential contribution of cold water to IBD development. These findings suggest that exposure to cold stress in the gut is also a novel risk factor for IBD in humans and a potential trigger for establishing experimental IBD in mice.

Dysregulation of the gut barrier in IBD is caused by environmental factors and genetic predisposition (Graham et al., 2020); however, identifying specific environmental factors has been difficult. Specific food additives are identified as environmental risk factors for IBD (Yang et al., 2018). Here, we aimed to identify whether the low temperature directly affects the development of colitis. Similar to observations in humans, the administration of cold water to mice attenuated gut transit; additionally, the mice exhibited a colitis-like phenotype of gut barrier injury. The fact that increased cold beverage consumption is related to some gastrointestinal symptoms seemingly suggests a link between cold stress and intestinal disorders. Indeed, our results indicate that exposure to cold water not only induces gut transit disturbances but also produces low-grade inflammation. In line with these considerations, cold stress exposure expectedly led to activation of ubiquitous inflammatory signaling pathways, suggesting local mucosal immune cell activation and immune cell trafficking. These are also the characteristics of IBD patients (Neurath, 2019).

Low-grade inflammation in colitis is associated with and may be promoted by gut microbiota dysbiosis (Frank et al., 2007). The low bacterial diversity and altered gut microbiota observed in the cold stress group in this study are extremely similar to those observed in IBD. For example, microbial diversity studies have demonstrated the overgrowth of Proteobacteria in IBD patients (Zhou et al., 2018). Under normal homeostasis, epithelial cells, tight junctions, and the local immune system prevent the translocation of pathogens in the gut. However, in genetically susceptible individuals, Proteobacteria expand to colonize the lumen and invade the lamina propria, further aggravating disruption of the gut barrier (Mukhopadhya et al., 2012). The host recognizes Proteobacteria *via* nucleotide oligomerization domain-like receptors, Toll-like receptors, and retinoic acid-inducible gene I-like receptors. Moreover, microbiota-derived products such as lipopolysaccharide, peptidoglycan, and flagellin from Proteobacteria trigger the activation of immune responses in the mucosa. Our study suggests that the increased abundance of pathogens accompanied by cold stress may contribute to gut barrier disruption in mice by accelerating inflammation in the gut.

Apart from the microbiota-derived products, the metabolites of the gut microbiota are also key actors in the development and exacerbation of IBD. Accumulating evidence suggests that signals from microbial metabolites affect mucosal integrity and immune homeostasis. Moreover, in IBD patients, the metabolites composition and function are disturbed seriously, including bile acids, short-chain fatty acids, and tryptophan (Lloyd-Price et al., 2019; Lavelle and Sokol, 2020). Significant alteration in the gut microbiota metabolites was also identified under cold stress exposure in this study. Interestingly, we found a remarkable reduction in the levels of several metabolites in the dopamine-related pathway.

Dopamine is a critical catecholaminergic neurotransmitter that present in the peripheral tissues and central nervous system simultaneously, which regulates blood pressure, sodium balance, glucose homeostasis, cognition, memory, the sympathetic nervous system, and mood (Pinoli et al., 2017). Although the dopamine is mainly synthesized in the brain, T cells, dendritic cells, and as well as by gut commensals such as members of the genus Clostridium (Vidal and Pacheco, 2020), recently, it has recently recognized as an important regulator of the immune system. Disturbance of the dopamine pathway affects both innate and adaptive immunity largely, causing the development of inflammatory pathologies (Vidal and Pacheco, 2020). A significant proportion of patients with gut barrier injury suffer from anxiety and depression (Byrne et al., 2017), implying a rational connection between the gut and the brain. Consistently, we examined their behaviors and found that cold stress leads to a tendency to depression. Our findings suggest that cold water stress results in reduced neuro-activities in gut microbiota and enhanced pro-inflammatory activity that promotes anxiety and depression, this is may be related to the dopamine pathway disturbance.

Nevertheless, our study has several limitations. We handled mice with cold water to explore the effect of cold stress to the gut barrier, microbiota, and metabolites. These findings acknowledge the important role of cold stress; however, it cannot mimic the condition of cold beverage consumption completely in humans because of the variety of beverages used and the differences between human and mouse physiology. In addition, although we find a significant overgrowth of pathogens and decreased dopamine-related metabolites in cold stress-treated mice, we cannot completely identify that the alteration in gut microbiota causes the gut barrier injury. Further studies to investigate the role of microbiota and their metabolites with the effect on gut barrier and behavior are also needed to deeply elucidate their causal or accompanied relationships. This is a novel attempt to describe the systematic and detailed phenotypes of this model, and the sample size is somewhat too small, more studies are needed to assure the conclusions furtherly.

## Conclusion

In summary, our study shows that exposure to cold stress promotes the development of colitis in mice. Our results may also have implications for human beings, as habit-forming consumption of cold beverages or food is implicated in IBD development.

## Data availability statement

The datasets presented in this study can be found in online repositories. The names of the repository/repositories and accession number(s) can be found at: https://www.jianguoyun.com/p/DRel6OYQuaiFChi1vOoEIAA.

## Ethics statement

The animal study was reviewed and approved by Animal Research Ethics Board of the Northwest University.

## Author contributions

JZ designed the study. LS, XW, YZ, and PL performed the research and wrote the manuscript. YH analyzed the data. CL and JL contributed the methods and models. All authors contributed to the article and approved the submitted version.

## Funding

This work was supported by grants from the National Natural Science Foundation of China (81900483), the Manned Spaceflight Advance Research Program (18032020103), Shaanxi Outstanding Youth Fund Project (2023-JC-JQ-65), and Air Force Medical University incubation pre-research program (2022-fhjsyxrc04).

## Conflict of interest

The authors declare that the research was conducted in the absence of any commercial or financial relationships that could be construed as a potential conflict of interest.

## Publisher’s note

All claims expressed in this article are solely those of the authors and do not necessarily represent those of their affiliated organizations, or those of the publisher, the editors and the reviewers. Any product that may be evaluated in this article, or claim that may be made by its manufacturer, is not guaranteed or endorsed by the publisher.

## Abbreviations

IBD: irritable bowel disease
IBS: inflammatory bowel syndrome
OTU: operational taxonomic unit
TST: tail suspension test
OFT: open field test
LC–MS: liquid chromatography-mass spectrometry
